# Microstructure and Isothermal Oxidation of Ir–Rh Spark Plug Electrodes

**DOI:** 10.3390/ma12193226

**Published:** 2019-10-01

**Authors:** Shifang Zhao, Jingyi Xia, Yimin Xia, Jianming Chen, Dekui Du, Huimu Yang, Jie Liu

**Affiliations:** 1 College of Mechanical and Electrical Engineering, Central South University, Changsha 410083, China; zhaoshifang@csu.edu.cn; 2 School of Materials Science and Engineering, Central South University, Changsha 410083, China; Xiajingyi@csu.edu.cn; 3 Zhuzhou Torch Spark Plug Co., Ltd., Zhuzhou 412001, China; Chenjianming@cntorch.com (J.C.); Dudekui@cntorch.com (D.D.); YANGHuimu@cntorch.com (H.Y.); liujie@cntorch.com (J.L.)

**Keywords:** Ir–Rh alloy, high-temperature oxidation, spark plug, spark plug electrode

## Abstract

High-temperature oxidation tests were performed on pure iridium, rhodium, and the iridium alloys, IrRh10, IrRh25, and IrRh40, at 1100 °C in a stable air environment for 60 h. The results of the oxidation were analyzed by X-ray photoelectron spectroscopy (XPS). Microstructural changes of the Ir–Rh alloys were characterized by scanning electron microscopy (SEM). XPS analysis results show that the main oxide of the Ir–Rh alloy in a 1100 °C environment was Rh_2_O_3_, and SEM analysis shows that the surfaces of the Ir–Rh alloys after oxidation formed both linear and ellipse-shaped corrosion pits, and had the same direction with the wire-drawing process. The oxidation behavior of Ir–Rh alloys, including the mass change, the reason for the mass loss, and the role of Rh in improving oxidation resistance performance, are discussed.

## 1. Introduction

As a new-generation engine, the turbo-charged direct injection gasoline engine has stringent requirements for spark plugs [1]. It demands that the spark plugs have more extended durability under harsh conditions, e.g., over 30 kV discharge impact, chemical attack by over 5 MPa pressure of fuel and combustion gas, a normal operating temperature up to 900 °C [2], and an ignition temperature that can reach 2300 °C [3]. Hence, to satisfy standard operation requirements and long-lifetime demands, spark plug electrodes must have excellent mechanical and chemical properties (e.g., high melting point, high strength, high electrical conductivity, and strong corrosion resistance) at high temperatures [4,5].

Before Ir and Ir–Rh alloys were used as an electrode material, Pt-based alloys (e.g., PtIr25, PtRh10) [6], as the central electrode of a spark plug, were used mostly for medium- and high-end engines. Although the performance of Pt-based alloys was qualified for the demands of new-generation engines [7,8,9], their application temperature, service life, and cost were still not optimal. Compared with platinum, iridium has the advantages of a higher melting point, higher strength, and better corrosion resistance [10]. Use of Ir and Ir-based alloys as spark plug electrodes have improved engine ignition performance, extended service life, and reduced costs. However, Ir, as a high melting point metal, has noticeable deficiencies in high-temperature oxidation resistance [11,12,13]. Ir can be rapidly oxidized or evaporated in an oxygen-enriched environment at high temperatures [14]. Since high-temperature oxidation resistance capacity is a crucial factor in improving spark plug performance [10,15], it is worthwhile to investigate the oxidation behavior of Ir–Rh alloys in the interest of minimizing the undesirable weight loss of alloys from Ir.

It has been reported that the main reason for weight loss is due to the formation of oxide species [10,16,17], and experiments have shown that the weight loss rate of iridium–rhodium alloys decreases with increasing rhodium content. However, to the best of our knowledge, no study has provided the reason that elemental Rh improves the oxidation-resistance capacity of Ir–Rh alloys [18,19]. Moreover, no relevant research has been completed to investigate the effect of deformation on high-temperature oxidation.

In this research, we evaluated the oxidation behavior of Ir–Rh alloy electrodes at 1100 °C under an air environment, and the effect of the wire-drawing process on high-temperature oxidation resistance of Ir–Rh alloys. To assess the actual high temperature anti-oxidation capacity of spark plug electrodes, all specimens were directly taken from an electrode production line (99.9% were supplied by Kunming Fullrolling Technology Development Co., Ltd., Kunming, China).

## 2. Experimental Details

High-purity elemental powders of Ir and Rh were used to prepare the alloys. After being compressed into cylindrical shapes, the compacted Ir and Ir–Rh alloys were smelted in a vacuum environment. To obtain the requested diameter for spark plugs, die-forging and wire-drawing processes were carried out on ingots. The alloy wires were wire-drawn multiple times. The amount of compression deformation was 5%–8% during each wire-drawing process, and when the total deformation reached 30%, the intermediate annealing treatment was carried out. Ultimately, the diameters of the alloy wires were compacted from 4 to 0.5 mm. The samples were then cut into wires of size Φ0.5 × 5 mm, which is the actual diameter of the spark plug electrode.

Samples, including pure Ir, Pure Rh, IrRh10, IrRh25, and IrRh40, were ultrasonically cleaned in ethanol and dried to remove surface impurities without damaging the original surface microstructure. Samples were placed in an alumina crucible measuring 200 mm × 100 mm × 20 mm in size, and the high-temperature oxidation tests for all samples were carried out in a muffle furnace in air at 1100 °C for 60 h. The mass change of oxidized samples was determined before and after oxidation using a 10^−4^ g sensitive electronic balance, and three samples were used for each composition. The final measurement result was the average of the testing values. The surface chemistry changes of samples were characterized by X-ray photoelectron spectroscopy (XPS), which was performed using a PHI-5000 VersaProbe II spectrometer (Ulvac-Phi, Inc., Kanagawa, Japan) using monochromatized Al Kα radiation (1486.6 eV). The residual pressure inside the analysis chamber was 10^−7^ Pa, and was calibrated by assuming the binding energy of carbonaceous carbon to be 284.8 eV. The microstructural changes in the alloys were characterized by scanning electron microscopy (SEM, SU3500, Hiatchi Corp., Tokyo, Japan) with an acceleration voltage of 15 kV. We used the secondary electron (SE) source only. For cross-sectional observation, the specimens after oxidation tests were embedded in epoxy, ground, and finely polished.

## 3. Results

### 3.1. Mass Change of High-Temperature Oxidation

This study focused on the final high-temperature oxidation resistance. Hence, the oxidation kinetics of samples were not evaluated. As shown in Table 1, weight loss experiments showed that the weight loss rate of samples decreased with increasing rhodium content. The mass change of Ir was more severe than observed in other work [17] under the same conditions. The weight loss of pure Ir in this test was over 50%, and Rh had a significant effect on the reduction of weight loss of the alloys. When the content of Rh reached 40%, the weight loss of the alloy was only 1.17% of pure Ir. Research has indicated that when the content of Rh is over 40%, the effect on improving the oxidation performance of Rh will decline [20].

### 3.2. Surface Chemical Changes of High-Temperature Oxidation

**Figure 1:**
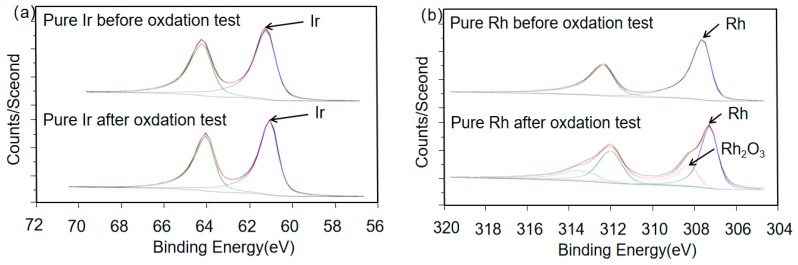
Ir 4f and Rh 3d spectra of (**a**) pure Ir and (**b**) pure Rh.

As shown in Figure 1a,b, the Ir 4f spectra of pure Ir before and after oxidation were almost the same. Table 2 lists the binding energy changes of the samples. The binding energy for the Ir 4f spectral region changed from 61.26 to 61.10 eV, and since the binding energy decreased, they were more likely to be the same substance, namely pure Ir [21]. The Rh 3h spectrum of pure Rh before and after oxidation is shown in Figure 1b, in which the binding energy of the Rh 3d spectral region before oxidation was 307.61 eV, which represents pure metal Rh. After oxidation at 1100 °C, the test results (Figure 1b and Table 1) were 307.28 and 308.24 eV, which indicates that in addition to the pure metal Rh, the reaction had formed Rh_2_O_3_ [22].

Table 2 shows the binding energy changes of all samples. The Ir and Rh in the alloys show similar results as those of the pure metallic elements. The binding energy after oxidation for Ir 4f was approximately 61.10 eV, and the two binding energies after oxidation for Rh 3d were 307.42 and 308.37 eV, respectively, corresponding to metallic Rh and Rh_2_O_3_, which were the only stable solid oxide species detected in this test.

### 3.3. Microstructural Characterization of High-Temperature Oxidation

**Figure 2:**
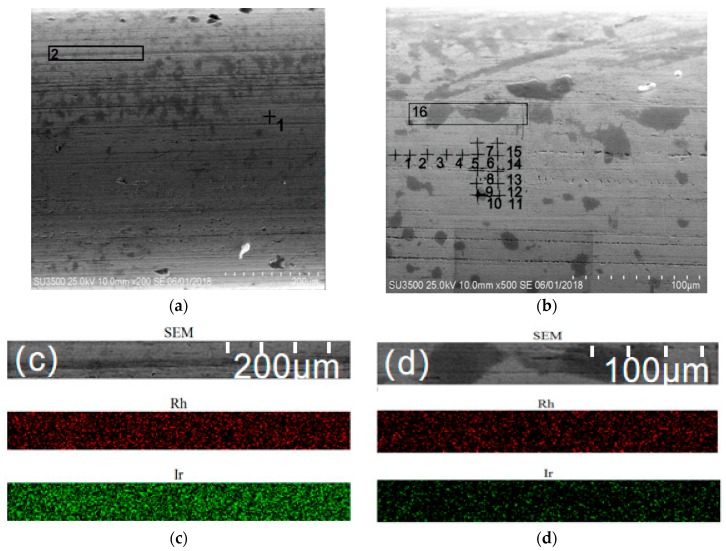
SEM images and electron-probe microanalysis (EPMA) maps of alloys before oxidation. SEM images of (**a**) IrRh10 and (**b**) IrRh25. EPMA maps of (**c**) IrRh10 and (**d**) IrRh25.

Figure 2a,b show the surface microstructure of IrRh10 and IrRh25 before the oxidation tests, respectively. There were noticeable machining scratches on the surface of the experimental samples, and the scratch direction was consistent with the axial direction. Moreover, numerous varisized pits were on the scratches, but no pattern was detected in the circumferential direction. To test the uniformity of the alloys, energy-dispersive spectrometry (EDS) was applied to analyze the IrRh25 surface. Figure 2b shows the analysis points of the EDS, and the distance between points was approximately 50 µm. The results are presented in Table 3, demonstrating that the Rh content did not exhibit an inhomogeneous distribution. Excluding the highest (29.5%) and lowest (26%) values, the average value was 27.34%. Although the surfaces of the alloys exhibited a slight inhomogeneity, no pattern was embodied. Electron-probe microanalysis (EPMA) was employed on both IrRh10 and IrRh25, and, as expected, the scanning results confirmed the uniformity of the alloys.

**Figure 3:**
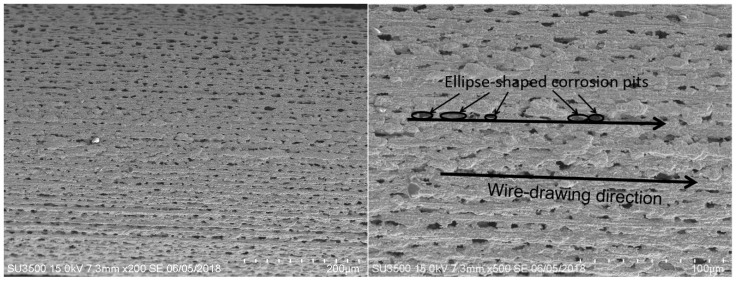
Surface morphology of IrRh10 after 60 h of high-temperature oxidation.

**Figure 4:**
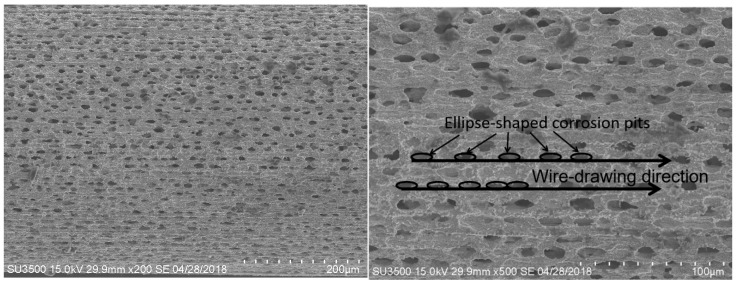
Surface morphology of IrRh25 after 60 h of high-temperature oxidation.

**Figure 5:**
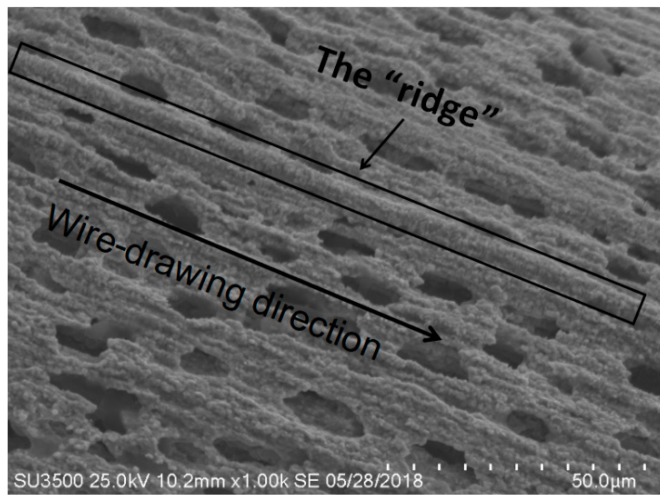
The “ridge” surface morphology of IrRh25.

After 1100 °C oxidation for 60 h, a porous structure of oxidation corrosion pits with linear permutations formed on both IrRh10 (Figure 3) and IrRh25 (Figure 4) in the same direction as the machining scratches. In particular, as shown in Figure 4, oxidation corrosion pits on the IrRh25 surface exhibited an approximately elliptical form. The average size of the elliptic oxidation pits was approximately 80–120 µm, as shown in Figure 5. The “ridge” surface morphology parallel to the axis was located on both sides of the oxidation pit arrays. In addition, scattered rhodium oxide particles were found on the IrRh25 surface at a higher magnification. The size of the IrRh10 oxidation pits were smaller compared with those of IrRh25 and presented wider spacing between pits. As shown in Figure 3, the “ridge” surface morphology on IrRh10 was lower and more obscured. The distance between pits on IrRh10 was wider than on IrRh25.

**Figure 6:**
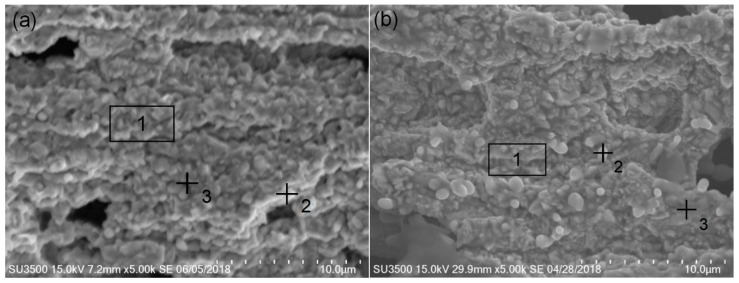
Surface energy spectra tests area and points of (**a**) IrRh10 and (**b**) IrRh25 after high-temperature oxidation.

The Ir–Rh alloys’ surface energy spectra results after oxidation are shown in Table 4, and the surface energy spectra tests area and points are shown in Figure 6. After oxidation, the distribution of surface elements varied greatly, the surface mass fraction of the Ir–Rh alloys was similar, and the mass fraction of Ir was significantly reduced. Correspondingly, the mass fraction of Rh increased. However, there were barely any oxidation surfaces on both Ir–Rh alloys. After oxidation, the surface could not be fully covered by rhodium oxides.

**Figure 7:**
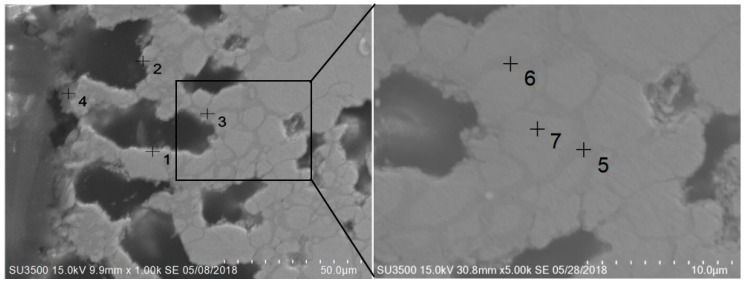
Cross-section of IrRh25 after oxidation.

As shown in Table 5, after oxidation, the average grain size was increased from the surface to the inside. Visible Rh segregation occurred on the grain boundary (see points 5 and 6 in Figure 7). The Rh content at the grain boundary reached 70%, and the width increased. There was no noticeable change inside the grain, indicating that the oxidation corrosion mainly began along the grain boundary but could not penetrate it.

## 4. Discussion

### 4.1. Oxide Formation

The primary oxidation product of Ir is IrO_3_ (g) [23], which is a highly volatile gas [16]. There are two opinions on the formation mode of IrO_3_ (g). Bao [17] suggests that IrO_3_ is formed in two steps. First, the reaction between iridium and oxygen can form solid IrO_2_, which can be further oxidized to IrO_3_. According to the previous study [24], IrO_3_ is directly formed by iridium and oxygen. However, according to Wimber’s standard-state free energy change equation, in the range of 1400–2400 K, this is true, therefore it is reasonable to infer that the free energy change for direct formation of IrO_3_ (g) was positive at a temperature of 1100 °C, and that the reaction could not be carried out. Hence, it was possible that the IrO_3_ gas was composed of another oxide (Bao [17] believes it should be IrO_2_ (s)). The results of our XPS tests (see Table 2) indicate that only pure iridium was detected after high-temperature oxidation, and no oxidized formation was detected. Since the decomposition temperature of iridium dioxide is approximately 1369 K [25,26], no solid iridium oxide species can stably exist at 1100 °C; they are either evaporated or decomposed to iridium and oxygen. Only very few solid iridium dioxide particles attached to the surface were formed during the temperature up-cooling process, and the IrO_2_ (s) formed from the temperature-rise period would be decomposed at 1100 °C. As a result, it is difficult to be detected by XPS. No matter how it occurred, only solid IrO_2_ could increase the mass after the oxidation process, and the decomposition of Ir may be due to the base metal.

The main oxides in the high-temperature oxidation process of Rh were RhO_2_ and stable Rh_2_O_3_. When the temperature was under 750 °C [27], Rh_2_O_3_ decomposed into RhO_2_ and oxygen. RhO_2_ continued decomposing into rhodium and oxygen when the temperature was higher than 1050 °C, but Rh_2_O_3_ stopped decomposing into RhO_2_ and oxygen. Since RhO_2_ was not detected by XPS, Rh_2_O_3_ was the main solid oxide formed at 1100 °C. However, the mass change of the Rh sample gained only 2.77% in mass, indicating that in addition to the amount of Rh_2_O_3_ that formed, a volatilization of the oxide existed to balance the weight gain. Carol and Mann [28] believe that the volatile oxide is Rh_2_O_3_.

### 4.2. Role of Rh

Owing to a lack of thermodynamic data for IrO_3_ (g) formed by other oxides, we cannot estimate the free energy change associated with the relative reaction. However, based on the mass loss ratio data from our test, we believe that the reaction should be very intense. If any IrO_2_ (s) formed, it would be decomposed into volatile IrO_3_ immediately. Hence, at the temperature of 1100 °C, the volatility of IrO_3_ and its evaporation contributed to the dominant mass loss in the oxidation test. The standard-state free energy change for IrO_2_ was determined by Jacob et al. [29], and can be represented by:

IrO_2_ ∆G_f_/J mol^−1^ = −239230 + 172.19T (±240).

Since the free energy change is almost proportional to temperature T, the free energy generation of IrO_2_ at 1373 K can be estimated as −2.81 kJ mol^−1^. Divergent data has been reported in the literature, but some of the data were positive [30]. For the Rh_2_O_3_ formation reaction, we have:

$$4\mathrm{Rh}+3O_{2}=2{\mathrm{Rh}}_{2}O_{3}.$$

The standard Gibbs free energy change can be represented by:

Rh_2_O_3_ ∆G_f_/J mol^−1^ Rh = −366365 + 282T (±120).

The negative value (−9.179 kJ mol^−1^) supports the fact that the reaction could proceed but not rapidly. The priority of Rh_2_O_3_ formation was only slightly higher than that of IrO_2_. Therefore, the element Rh in the Ir–Rh alloy could not perform well in terms of its ability to absorb oxygen, such as Al in an IrAl alloy, which means that the free energy change of aluminum oxide was much lower than that of iridium oxide. The only explanations are that Rh_2_O_3_ was relatively stable compared with iridium oxide, and that the segregation of Rh at the grain boundary of the alloy increased the contact area between Rh and oxygen, thus playing a role in improving the oxidation resistance of the Ir–Rh alloy.

### 4.3. Effect of the Wire-Drawing Process

**Figure 8:**
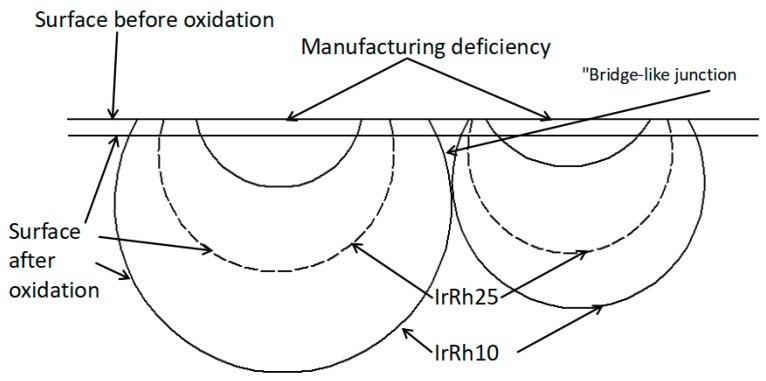
Schematic of oxidation-pit development.

**Figure 9:**
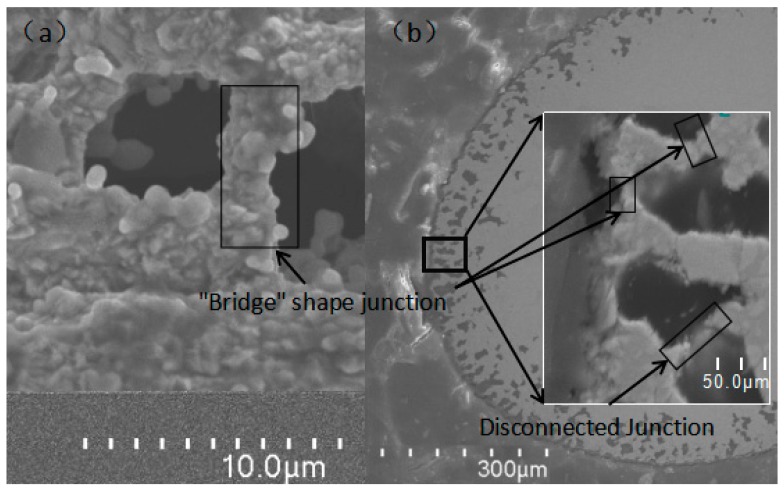
"Bridge" structure on IrRh25 surface after oxidation. (**a**) “Brigde” shape junction; (**b**) Disconnected junction.

Compared with other researchers’ experimental results, the mass loss results from our test were much higher. The surface defects on the samples caused by the wire-drawing process were likely the primary reason. As shown in Figure 2, numerous grooves and pits on the surface were in the same direction as the wire-drawing process. As demonstrated in Figure 8, the oxidation started from the location of the pit and the grooves, and then gradually expanded to the surrounding areas, thus increasing the exposed surface area and accelerating the oxidation rate. Therefore, an oxidation pit was formed in the same direction as the wire-drawing process. With further oxidation, the oxidation pit was connected internally, forming a "bridge-like junction zone," as shown in Figure 9.

**Figure 10:**
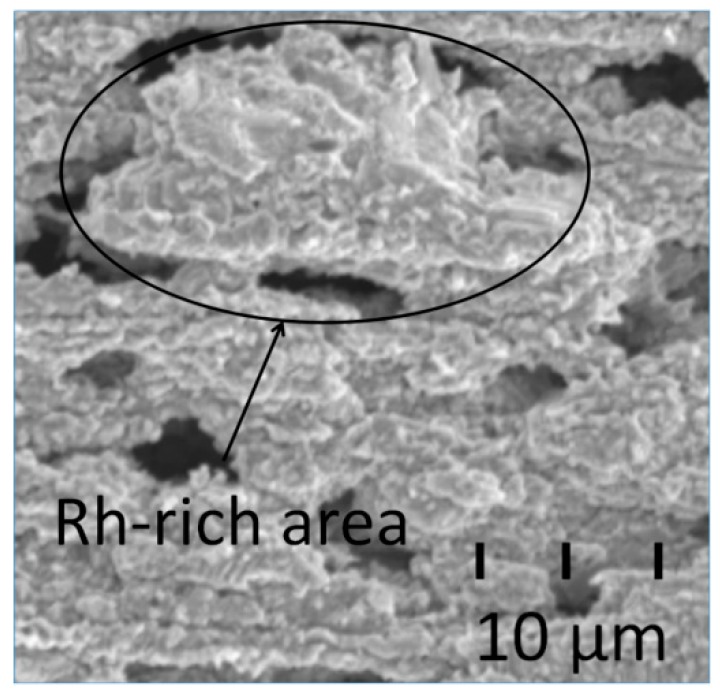
IrRh10 surface after oxidation, showing a loose oxidized particle.

Another possible reason for abnormal weight loss was the segregation of elemental Rh, as there were some Rh-rich areas that could form stable particles encased by Rh_2_O_3_, as indicated in Figure 10. As the oxidation continued, although the particles themselves were not oxidized further, the surrounding particles were continuously oxidized, which finally led to the isolation of the particles and their detachment from the alloy. According to the energy spectrum analysis results presented in Table 4, after being oxidized for 60 h, the surface still had an area of approximately 60% Ir, which indicated that the area was barely oxidized after the isolated particles were detached from the alloy.

## 5. Conclusions

In this paper, the Ir oxidation behaviors of IrRh10, IrRh25, IrRh40, and Rh under an environment of 1100 °C were analyzed. Studies have shown that the primary weight loss of the Ir–Rh alloys were via the loss of iridium oxides. Although the addition of Rh elements can improve the oxidation resistance, elemental Rh does not have an affinity for oxygen because the standard free energies of oxide formation for Ir and Rh are similar. Because of the wire-drawing process, the surface defects caused by this process increased the oxidation rate, resulting in a large number of regular oxidation pits that were accompanied by the shedding of oxidized particles, finally leading to an abnormal weight loss rate. Further research is needed to prove the detachment mechanism using a mathematical method, and future work should investigate the critical value of detachment.

## Figures and Tables

**Table 1:** Mass change of pure elements for 60 h under 1100 °C oxidation.

Composition	Before (g)	After (g)	Mass Change (g)	Weight Loss Ratio (%)	Mass Change (mg/mm^2^)
Pure Ir	0.2397	0.111	0.1287	53.69	1.498
IrRh10	0.2340	0.1968	0.0404	15.90	0.414
IrRh25	0.2417	0.219	0.0227	9.39	0.22
IrRh40	0.1266	0.1258	0.0008	0.63	0.013
Pure Rh	0.0989	0.1016	−0.0027	−2.73	−0.042

**Table 2:** Peak values of X-ray photoelectron spectroscopy (XPS) test results before and after oxidation tests.

Composition	Atomic Concentration before Test (%)	Atomic Concentration after Test (%)	Binding Energy before Test (eV)	Binding Energy after Test (eV)
Pure Ir			Ir: 61.26	Ir: 61.10
Pure Rh			Rh: 307.61	Rh: 307.2, Rh_2_O_3_: 308.24
IrRh10	Ir: 83.25	Ir: 46.42	Ir: 61.41	Ir: 61.08
	Rh: 16.75	Rh: 53.58	Rh: 307.66	Rh: 307.42, Rh_2_O_3_: 308.37
IrRh25	Ir: 63.22	Ir: 38.19	Ir: 61.13	Ir: 61.10
	Rh: 36.78	Rh: 61.81	Rh: 307.69	Rh: 307.46, Rh_2_O_3_: 308.41
IrRh40	Ir: 44.11	Ir: 32.04	Ir: 61.02	Ir: 61.07
	Rh: 55.89	Rh: 67.96	Rh: 307.56	Rh: 307.38, Rh_2_O_3_: 308.33

**Table 3:** Energy-dispersive spectrometry (EDS) analysis results of IrRh25.

Element	Test Points(wt.%)														
	1#	2#	3#	4#	5#	6#	7#	8#	9#	10#	11#	12#	13#	14#	15#
Rh	28.23	28.62	28.09	27.1	29.5	28.88	26.9	26.57	27.27	26.0	28.17	26.41	26.48	27.47	27.22
Ir	71.77	71.38	71.91	72.9	70.5	71.12	73.1	73.43	72.73	74.0	71.83	73.59	73.52	72.53	72.78

**Table 4:** Surface mass fraction of alloys after oxidation.

Element	IrRh10 (wt.%)			IrRh25 (wt.%)		
	1#	2#	3#	1#	2#	3#
O	10.484	26.979	7.352	6.343	7.331	5.949
Rh	63.978	52.988	32.458	60.974	56.64	32.849
Ir	25.537	20.033	60.19	32.683	36.029	61.203

**Table 5:** EDS analysis results of IrRh25 after oxidation at cross-section.

Element	Test Points (wt.%)						
	1#	2#	3#	4#	5#	6#	7#
O	5.158	2.439	0.396	23.228	0	0	0
Rh	27.826	59.068	66.66	39.461	68.066	70.018	23.352
Ir	67.016	38.493	32.945	37.311	31.934	29.982	76.648
